# Optical Microscopy as a Tool for Assessing Parenteral Nutrition Solution Stability: A Proof of Concept

**DOI:** 10.3390/ph17101330

**Published:** 2024-10-05

**Authors:** Luis Otero-Millán, Brais Bea-Mascato, Jose Luis Legido Soto, María Carmen Martín de la Cruz, Noemi Martínez-López-De-Castro, Natividad Lago-Rivero

**Affiliations:** 1 Pharmacy Department, University Hospital Complex of Vigo, 36312 Vigo, Spain; 2 NeumoVigo I+i Research Group, Galicia Sur Health Research Institute (IIS Galicia Sur), SERGAS-UVIGO, 36312 Vigo, Spain; 3 Innovation in Clinical Pharmacy Research Group (i-FARMA-Vigo), Galicia Sur Health Research Institute (IIS Galicia Sur), SERGAS-UVIGO, 36312 Vigo, Spain; 4 Applied Physic Department, Faculty of Sciences, University of Vigo, 36310 Vigo, Spain

**Keywords:** parenteral nutrition, physicochemical stability, precipitation, lipid emulsion

## Abstract

**Background/Objectives**: Parenteral nutrition (PN) is used when enteral feeding is not possible. It is a complex mixture of nutrients that must meet a patient’s needs but can face stability issues, such as lipid emulsion destabilisation and precipitate formation. Stability studies are complex, and the methodologies used are very varied in the literature. In addition, many studies are outdated and use outdated components. This study conducts a stability analysis of PN solutions using optical microscopy. **Methods**: Samples were prepared according to clinical practice standards and previous studies. We used a counting chamber for optical microscopic observations and different storage conditions (RT, 4 °C 1–14 days). **Results**: Precipitates larger than 5 µm were found in 8 out of 14 samples after 14 days of storage at room temperature, and none were observed in refrigerated samples. More lipid globules larger than 5 µm were detected in samples stored at room temperature than in those stored in a refrigerator after 14 days. Additionally, the number of large globules generally increased from day 1 to day 14 in most samples. **Conclusions**: The observed precipitates were probably calcium oxalate crystals, the formation of which is possible in PN but is not expected under the usual storage conditions in a hospital environment. Prolonged storage time and storage at room temperature increases the formation of these precipitates. These findings highlight the importance of using filters during both the preparation and administration of PN to prevent large particles from reaching patients.

## 1. Introduction

In a number of clinical situations, feeding patients is made difficult by the inability to use the enteral route [1]. In these cases, parenteral nutrition (PN) is the common alternative used to feed these patients. PN is a mixture whose composition must cover a patient’s macronutrient and micronutrient requirements [2]. For this reason, it is a complex mixture (comprising amino acids, lipids, carbohydrates, electrolytes, vitamins and trace elements), in which a multitude of physicochemical interactions can occur. Currently, it is common for PNs to be prepared as “all-in-one mixtures” (mixing amino acids, carbohydrates and lipids), resulting in an emulsion. This is the main reason why PNs present serious stability problems that can have a negative impact on patient safety [3,4].

The potential destabilisation of the lipid emulsion is one of the main risks in these mixtures. Lipid globules begin to coalesce into larger globules until phase separation occurs [5,6].

Another risk classically studied in the scientific literature is precipitate formation. The main concern at the beginning of PN use has been calcium phosphate precipitation. The mechanism of the formation of these precipitates is explained by the different ionisation states that can be formed by the phosphate group of the salts that are commonly added to the PN as a phosphorus supply [7,8,9,10,11,12]. These phosphorus ions react with the calcium cations, forming the precipitate. It is a process influenced especially by pH (favoured at high pHs) but also by other factors such as time, temperature or even other species in the solution (amino acids). The recommendations used in daily practice establish the maximum concentration limit for calcium and phosphorus (Calcium (mEq/L) + phosphate (mMol/L) ≤ 30 mEq/L), although there are specific ranges depending on the type of phosphorus salt (inorganic/organic) and the concentration of amino acids [13].

Over time, more stable compounds in solution have become commercially available, such as organic salts of both phosphorus and calcium, which are compatible at higher concentrations [9,14,15,16,17]. In any case, there are studies that also show the formation of precipitates using these products [9,14,18,19].

In addition, the literature shows the formation of precipitates with several compositions. Some trace elements can interact with amino acids to form insoluble species. This is the case for sulphur-containing amino acids (cysteine and methionine), which can be affected by reactions with elements such as zinc, copper, iron and selenium [20]. Another case is the formation of calcium oxalates. Oxalic acid is a degradation product of ascorbic acid, a common component of PN [12,13], which can react with the calcium also present in PN.

The approaches followed in the stability studies of PN mixtures are varied and scattered, suggesting that there are no standards to date. A large part of the literature focuses on lipid emulsion stability, where the aim is to obtain globule size distributions in order to assess compliance with the limits established in the different guidelines or pharmacopoeias [21,22]. Other studies focus on analysing precipitate formation. In these studies, microscopy or spectroscopy techniques are more commonly used to characterise the precipitate formed in terms of its shape and elemental composition [18,23,24,25,26]. Optical microscopy has been used in studies assessing the stability of PN by simple observation [8,27], using counting chambers [26] or even looking for correlations with more precise laser techniques [28]. In addition, the European Pharmacopoeia also includes information on the optical microscopic analysis of pharmaceutical products [29].

It is important to use accurate and reliable methods to determine the stability of the PN. Therefore, optical microscopy is used to analyse both the stability of the lipid emulsion and the formation of precipitates. It is not possible to calculate size distributions or similar parameters with this technique, but it is equally used in this type of study and can complement data from other techniques.

Due to the extremely complex composition of PN, its stability studies are very complex. It would be useful to have updated stability data, with new components used, including as many stability-related variables as possible and using different analytical techniques.

In this study, we performed a stability analysis of PN solutions using optic microscopy.

## 2. Results

### 2.1. Precipitate Formation

**Figure 1:**
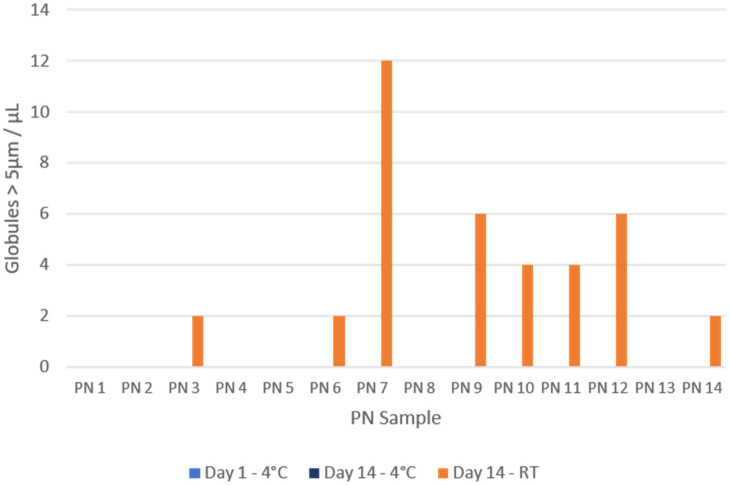
Precipitates with a size greater than 5 µm per µL of sample. All days of analysis (1 and 14) and both storage conditions are represented (RT: room temperature; 4 °C: refrigerator).

**Figure 2:**
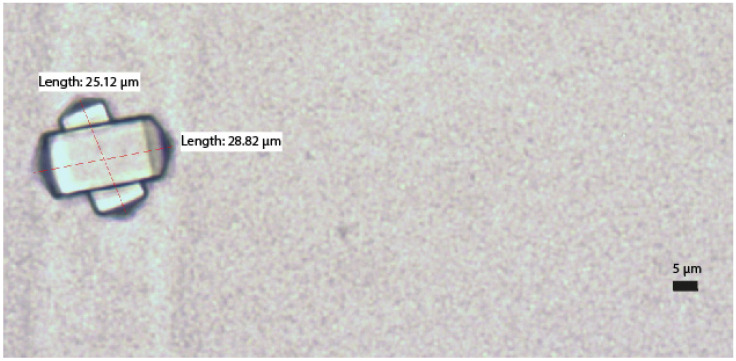
Example of precipitate detected in the analysis.

The results for precipitates with a diameter greater than 5 µm, expressed as precipitates/µL after conversion according to the counting chamber manufacturer’s indications, are summarised in Figure 1. Precipitates were observed in 8/14 samples. All precipitates corresponded to samples analysed after 14 days of storage at room temperature, with none in the samples stored in the refrigerator. An example of the precipitates found is shown in Figure 2.

### 2.2. Globule Size

**Figure 3:**
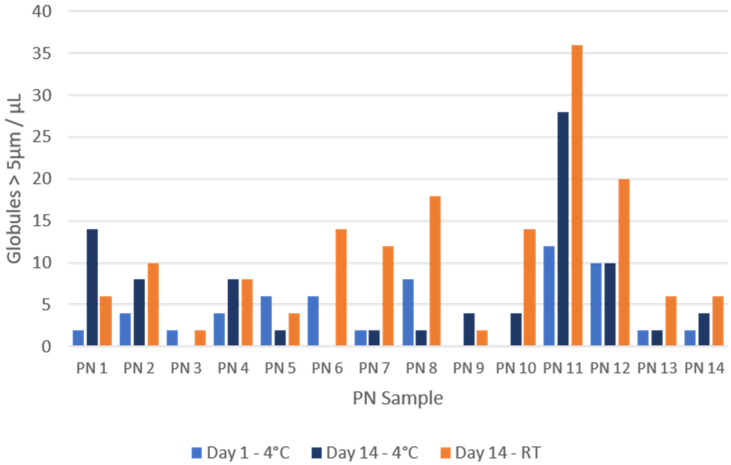
Globules with a size greater than 5 µm per µL of sample. All days of analysis (1 and 14) and both storage conditions are represented (RT: room temperature; 4 °C: refrigerator).

Figure 3 shows the results obtained for lipid globules larger than 5 µm, expressed as globules/µL after the conversion indicated by the counting chamber manufacturer.

**Figure 4:**
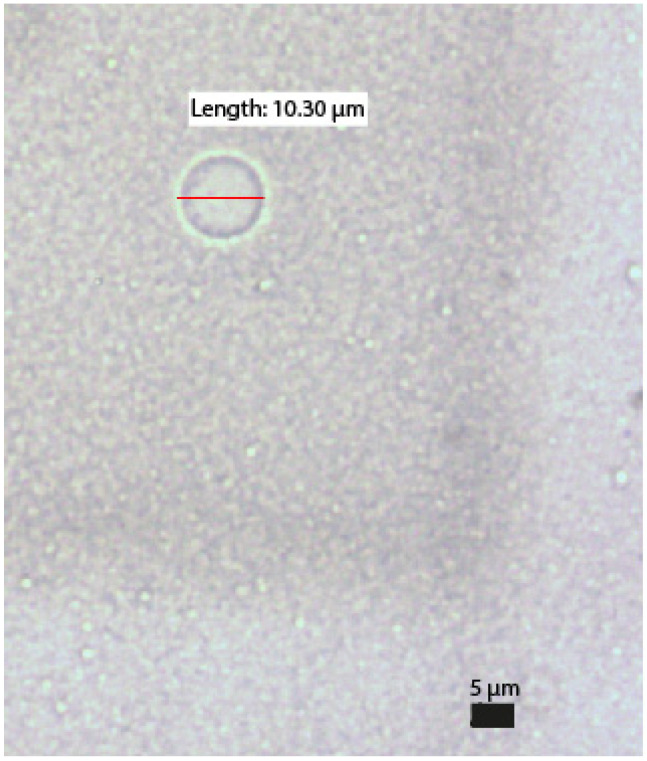
Example of a globule quantified in the analysis.

In 11/14 of the samples, the number of globules > 5 µm detected at room temperature was higher than that detected in the fridge after 14 days of storage. Only in sample PN1 and PN9 were more globules greater than 5 µm measured in the 14-day fridge sample, while PN4 contained the same number of globules on both temperatures. Similarly, in 12/14 samples, the number of globules larger than 5 µm at 14 days was higher than that detected on day 1. Figure 4 shows an example of one of the globules found.

## 3. Discussion

Optical microscopy (OM) was used to visually examine the samples based on a count of globules and precipitates larger than 5 µm. The results showed the existence of large globules and precipitates (above 5 µm in size) that are potentially dangerous for patients receiving PN, as their size can obstruct the microvasculature and cause pulmonary or cerebral embolisms. Increased oxidative stress in the lungs and liver dysfunction have also been reported, especially severe in neonates, potentially leading to growth retardation [3,4,30,31,32].

The use of OM is widely represented in PN stability studies. As noted in the scientific literature, different methods can be used to analyse stability; for example, laser light scattering, photon correlation spectroscopy, electrical zone detection, light obscuration, etc. USP Chapter 729 recommends dynamic light scattering (for mean droplet diameter) and light obscuration (for the large diameter fraction of the droplet size distribution) [21,22,33,34,35]. These methods offer accurate and reliable results; however, Hospital Pharmacy Services often do not have the necessary equipment. OM is an often readily available method that is easy to use and does not require sample preparation with dilutions or other manipulations, which can be an advantage as it does not alter the PN. The detection of precipitates by OM ensures that they are formed during storage within the PN and do not involve crystallisation processes caused by the evaporation of the sample [36,37]. We did not observe any decrease in the volume of the counting chamber during the observation, so we excluded the possibility of evaporation.

However, there are limitations. The use of OM is a more laborious technique compared to the use of automated equipment and it does not allow for the measurement of submicrometric particles and, consequently, complete size distributions are not available for the calculation of the mean globule size and the fraction of large globules above 5 µm (USP control parameters). In addition, it also has high subjectivity in its measurements. In this sense, the work of Driscoll et al. [28] is interesting. They studied the possibility of assigning limits based on measurements made with OM by comparing them with globule size data measured with photon correlation spectroscopy or DDL and LE/SPOS.

A similar study to ours was the work of Janů et al. [26], where they evaluated the stability of PN solutions with globule size measurements using a camera-equipped OM and the same FastRead Biosigma^®^ counting chamber as was used in our study. Solution composition was calculated according to centre protocols, varying the sources of lipids (Lipoplus^®^ and Smoflipid^®^) and the amounts of electrolytes. Samples were kept at room temperature on the first day and then stored for 30 days in a refrigerator. Measurements were conducted on days 2, 5, 8 and 30. They observed that the count of globules larger than 5 µm tended to increase over time and correlated more strongly with higher calcium concentrations and processing with Lipoplus^®^. In our samples, we found a lower number of globules larger than 5 µm than reported by Janu et al. [26]. In their study, 6/16 Lipoplus^®^ samples exceeded 50 globules/µL at day 8 (the closest day to the one used by us). In our study, only 3/14 samples exceeded 15 cells/µL. Their study discusses a possible relationship between calcium concentration or a high CAN and an increased number of large particles. For their samples, the CAN was calculated to be between 364 and 1247 mmol/L. All our samples have a higher value (CAN = 2020–4900 mmol/L), so it does not seem that the differences are due to this factor. The rest of the compositions were different (source of amino acids), so the reasons for the existence of these differences in the blood cell count could be due to multiple causes, such as subjectivity in the measurement or the shorter analysis time in our study (day 14 vs day 30). Future studies using OM would require a larger number of samples with different concentrations in order to find a relationship between composition and instability.

Another work using OM was that of Ribeiro D. de O. et al. [8]. In this case, an optical microscope was coupled to software for image processing and globule counting. The results were grouped according to the following size intervals: 0–1 μm; 1–2 μm; 2–3 μm; and >3 μm. They observed that the samples remained stable because none of the lipid globules exceeded 4 μm in any of the mixtures during the seven days of analysis at any temperature, even in the presence of high amounts of calcium. Again, methodological changes between the studies, such as different sample storage times, compositions or subjectivity in their measurements, may be responsible for the observed differences.

Regarding the modified conditions in our study, we observed a clear influence of storage time and temperature on the appearance of globules and precipitates larger than 5 µm, increasing their number after more days of storage at room temperature. This was particularly relevant in the case of precipitates, where they were only observed in samples stored at room temperature and never on the first day of analysis (only after 14 days). We only have data from day 1 and day 14, so we cannot deduce from which day they start to form. It would be necessary to extend the study with more intermediate control points.

These are classical factors that have been widely studied in the field of PN stability. Storage at room temperature and long storage times are detrimental in terms of stability [13,38]. Our findings are consistent with theoretical expectations and are aligned with previous studies on PN stability. These results highlight the importance of carefully considering storage factors to maintain PN stability.

The different compositions of the samples is another variable that may be responsible for the results obtained. We have observed that the sample with the highest number of precipitates detected was the sample with the highest phosphorus concentration. This observation is consistent with expectations, since most of the information on precipitation in PN is available for calcium phosphate. Its formation has always been a major concern, especially with the use of inorganic salts in PN preparation, because of the increased risk of precipitation. With the introduction of organic salts of phosphorus (sodium glycerophosphate and glucose-1-phosphate) and calcium (calcium gluconate), calcium phosphate precipitation decreases [17]. In our study, we used organic calcium and phosphate salts for the preparation, but we detected precipitates as well. However, this is in accordance with previous studies where precipitation was observed despite the use of organic salts [14,19].

As for the characterisation of the precipitates found, we only have information on the shape of these precipitates. In our study, all the precipitates observed were identical (rectangular prism with pyramidal base). In another previous study by our group using electron microscopy, similar precipitates were identified and characterised as calcium oxalate precipitates. In this case, the information obtained was the shape again (identical) but also the composition (calcium, carbon and oxygen) [37]. Other similar studies also show this type of precipitate in PN [36,39]. Ascorbic acid is the least stable vitamin, and its degradation leads to a final product which is oxalic acid. This degradation is mediated by oxygen, which can come into contact with the PN at the time of preparation [12].

Continuing with the composition, the next group of samples with the highest number of precipitates are the samples with decreased lipid concentrations. Phospholipids can interact with calcium ions to form complexes, causing calcium to be “sequestered” and preventing its participation in precipitation processes. Similar studies using organic phosphate salts show more precipitates in samples without lipids [18].

In line with this finding, a higher globule count is also observed in samples with a lower lipid concentration compared to other samples. Although the literature indicates a consensus and provides guidelines that a low lipid concentration is negative for stability [13], there are no published results in relation to this assertion. Currently, lipid sources based on long-chain fatty acids are not used due to their lower stability and the existence of new formulations that are more stable and have more beneficial lipid profiles for patients [33].

These observations are consistent with our previous studies, where we have analysed in detail the processes of lipid aggregation and coalescence and their possible causes. Dynamic light scattering measurements were made and higher percentages of larger globules were also observed in samples with lower lipid and amino acid concentrations [40]. The conclusions we can extract from compositional changes are limited in this study. We have a small number of samples, which is insufficient to make strong conclusions. It would be necessary to extend our study using a larger number of samples and a greater variety of concentrations in the components where we observed tendencies towards instability.

## 4. Materials and Methods

### 4.1. Sample Preparation

Sample preparation followed the same procedures used to prepare samples in daily clinical practice and has already been described in previously published studies by this group [40]. A total of 23 samples were originally prepared in a larger study [40], of which only 14 were analysed by light microscopy. Table 1 shows the composition of the 14 samples analysed in this study. Glucose, potassium and calcium concentrations were the same in all samples (75.00 g/L, 60.00 mMol/L and 25.00 mMol/L, respectively). We used organic calcium and phosphorus salts.

The preparation process adhered to the standards and procedures for area cleaning and disinfection, the use of aseptic techniques, laminar flow cabinets (Telstar BH100^®^, Barcelona, Spain) and the evaluation of the final product. The preparation followed the latest Spanish consensus on the preparation of PN mixtures, established in 2008 by the Working Group on Artificial Nutrition Pharmacy of the Spanish Society of Parenteral and Enteral Nutrition (SENPE) [13]. A 500 mL stock solution was prepared and then divided into two portions: 250 mL was stored at room temperature (RT) and the other 250 mL was stored in a refrigerator at 4 °C. The sample was aliquoted into 50 mL polypropylene syringes (air-free, with luer-lock caps) from the original EVA bag, and these were kept for 14 days. For the analysis, the required volume was withdrawn from the syringes. Throughout the process, measures were taken to protect the samples from light exposure. Sterile materials were consistently used to prevent microbiological contamination, and the preparation was carried out in a sterile environment within laminar flow cabinets.

### 4.2. Optical Microscopy

We have analysed the samples with optical microscopy to evaluate their stability, looking at globule size and the appearance of precipitates. Measurements were taken at different times and under different storage conditions. For this purpose, a Fast Read Biosigma^®^ (Venecia, Italia) counting chamber was used. Although this chamber was designed for urine analysis (cell counting), it has been used in stability studies of PN samples similar to ours [26]. It is a slide-type chamber, protected by a transparent film, with 10 independent chambers containing a standard volume of 7 µL. The sample is deposited by capillary action and distributed homogeneously throughout the chamber. The chamber has 10 independent chambers or a “Grid”. Each contains 10 divisions, called “Squares”, which are divided into 16 subdivisions, called “Sectors”. The results are shown as droplets per µL after the following conversion according to the manufacturer’s instructions:

(1) Droplets/µL=(∑Droplet count in a Square)×10/N

where *N* is the number of “*Squares*” counted (in our case, 5). Our sample did not require any dilution or preparation, so wat is not necessary to use a dilution factor.

On the day of preparation, after preparing each sample, 10 mL syringes were loaded. Sterile syringes were used, which were purged to remove air and closed with a sterile luer-lock cap. Each syringe was then labelled and protected from light. The syringes were stored in a refrigerator until the next day. At day +1, visualisation was performed by taking the aliquot needed to load the counting chamber from any of the samples (up to this point they were considered equal). After the first day’s analysis, the syringes were stored again in a refrigerator or at room temperature, as appropriate, for the next analysis on day 14. A Nikon Eclipse 50i^®^ (Melville, NY, USA) optical microscope with image analysis was used. Images were saved and analysed with the 40× objective. Only precipitates with a clearly crystalline form were quantified. Precipitates/structures with amorphous forms were not considered.

## 5. Conclusions

The observed precipitates were probably calcium oxalate crystals, the formation of which is possible in PN but is not expected under the usual storage conditions in a hospital environment. Prolonged storage time and storage at room temperature increase the formation of these precipitates.

High cation concentrations (high CAN) have not been associated with increased lipid emulsion instability and precipitate formation.

The use of organic calcium and phosphate salts, together with the other preparation conditions used, seems to prevent the formation of calcium phosphate precipitates or their derivatives.

Our results highlight the importance of the use of filters during the preparation and administration processes to avoid the possible transfer of large particles to patients.

## Figures and Tables

**Table 1:** Details of the modifications made to the sample formulations used in this study. Calculation of CAN and OSM is included.

Sample	N (g/L)	Prot (g/L)	Lip (g/L)	Na (mMol/L)	Mg (mMol/L)	P (mMol/L)	OSM (mOsm/L)	CAN (mMol/L)
PN1	4.00	25.00	20.00	40.00	25.00	12.50	1110.00	3300.00
PN2	4.00	25.00	20.00	40.00	37.50	12.50	1160.00	4100.00
PN3	4.00	25.00	20.00	40.00	50.00	12.50	1210.00	4900.00
PN4	4.00	25.00	20.00	40.00	5.00	20.00	1043.50	2020.00
PN5	4.00	25.00	20.00	48.00	5.00	30.00	1101.50	2028.00
PN6	4.00	25.00	20.00	80.00	5.00	50.00	1217.50	2060.00
PN7	4.00	25.00	20.00	120.00	5.00	100.00	1507.50	2100.00
PN8	4.00	25.00	15.00	40.00	5.00	12.50	1015.00	2020.00
PN9	4.00	25.00	10.00	40.00	5.00	12.50	1000.00	2020.00
PN10	4.00	25.00	5.00	40.00	5.00	12.50	985.00	2020.00
PN11	4.00	25.00	2.50	40.00	5.00	12.50	977.50	2020.00
PN12	4.00	25.00	1.24	40.00	5.00	12.50	973.75	2020.00
PN13	3.50	21.87	20.00	40.00	5.00	12.50	995.63	2020.00
PN14	3.00	18.75	20.00	40.00	5.00	12.50	961.25	2020.00

PN1–PN14: PN samples; N: nitrogen (Aminoven Infant 10% Fresenius Kabi^®^, Barcelona, Spain); Prot: protein; Lip: lipids (Lipoplus 20% Braun^®^, Melsungen, Germany); OSM: osmolarity; CAN: critical aggregation number, calculated according to cation concentration to analyse its relationship to stability (CAN = a + 64 b + 729 c, where a, b and c are the sum of the concentrations (mmol/L) of mono-, di- and trivalent cations, respectively [41]). Other components used: Glucose 50%, Grifols^®^, Barcelona, Spain; Sodium Chloride 20%, Braun^®^; Potassium Acetate 1M, Braun^®^; sodium glycerophosphate (Glycophos Fresenius Kabi^®^); calcium gluconate (Suplecal Braun^®^); and Magnesium Sulfate 15%, Genfarma^®^, Madrid, Spain. Vitamins: Vitalipid Fresenius Kabi^®^. Trace elements: Meinsol Oligo-zinc Fresenius Kabi^®^ and water for injection (Grifols^®^).

## Data Availability

Data is contained within the article.
